# Triboluminescence of Centrosymmetric Lanthanide β-Diketonate Complexes with Aggregation-Induced Emission

**DOI:** 10.3390/molecules24040662

**Published:** 2019-02-13

**Authors:** Ho-Yin Wong, Wesley Ting Kwok Chan, Ga-Lai Law

**Affiliations:** Department of Applied Biology and Chemical Technology, Hong Kong Polytechnic University, Hung Hom, Kowloon 999077, Hong Kong, China; hy-chris.wong@connect.polyu.hk (H.-Y.W.); wesley.chan@polyu.edu.hk (W.T.K.C.)

**Keywords:** triboluminescence, lanthanide, β-diketonate

## Abstract

Triboluminescence (TL) is a form of light emission induced upon mechanical forces on the material. However, our understanding of this phenomenon is still unclear and more examples are therefore needed in order to elucidate its mechanism. In this work, two types of TL complexes, [Eu(*pp*-dbm-Cl_2_)_3_phen] and [Eu(*mm*-dbm-Cl_2_)_3_phen], which also displays aggregation-induced emission (AIE) were synthesized and investigated for its photo-physical and crystal structural properties. These complexes were crystallized in a centro-symmetric space group *P*2_1_/*n*, and remarkably, displayed TL upon grinding that may be due to the presence of extensive π···π, C-H···π and C-H···Cl-C interactions in the close molecular packing of its structure. This rare example deviates from the widely accepted mechanism of TL, hence widening the scope of our understanding in the area.

## 1. Introduction

Triboluminescence (TL) is a form of luminescence induced by applying mechanical force to crystalline materials [1]. It has attracted considerable attention because of its potential application such as pressure sensing [2]. Even though this optical phenomenon has been discovered more than a century ago, the understanding of its mechanism is still elusive. It has been widely accepted that the piezoelectric effect of non-centrosymmetric crystals causes TL because piezoelectric charges can excite the molecules. However, some examples of structures with centrosymmetric space groups such as [Eu(tta)_3_bipy], [Eu(tta)_3(_dmbp)].H_2_O, dmpy[Eu(tta)_4_], mlq[Eu(tta)_4_], apy[Eu(tta)_4_], and [Tb(AP)_6_]I_3_ displaying vibrant TL have refuted this correlation [3,4,5,6,7], in fact, there are many influential factors such as ionic charge, crystal defects, gases environment, and pressure [8]. To study this phenomenon, lanthanide(III) chelates are ideal candidates due to their large Stoke shift that can eliminate inherent reabsorption problems, especially associated to organic compounds [9,10].

Molecular packing is also known to be a crucial factor in affecting various optical phenomena and is often neglected. Only in recent years has there been more studies directed in this area with examples of organic luminogens showing aggregation induced emission (AIE) and TL. Such work has brought insight into the area, where the influence of the crystal packing also has a dominant role in parallel with the electronic structure of the molecule [11,12]. For example, it has also been shown that the para or meta position of phenanthrol [9,10-*d*]imidazole (PI) group on tetraphenylethene (TPE) can lead to a difference in TL. While both crystals of *mm*-TPE(PI)_2_ and *pp*-TPE(PI)_2_ are centrosymmetric, the former, with stronger intermolecular interactions and higher dipole moment, is TL-active, but the latter is not [13]. As the mechanism of TL of different materials can vary, the study of an AIE-active compound with a centrosymmetric structure can bring insight into developing structural and optical correlations to TL, especially by examining the intermolecular interactions in the molecular packing.

However, designing AIE-active lanthanide(III) chelates are still challenging due to the controversial mechanism of AIE [14]. The key factor is the restriction of intramolecular rotation and intermolecular π-π stacking. Exceptional cases have also been explained by molecular planarization, intramolecular charge transfer, twisted intramolecular charge transfer, E-Z isomerization, and J-aggregate formation. Lately, there have been reports of AIE-active transition metal complexes caused by the presence and formation of halogen bonds (XB) in the structure [15].

**Figure 1:**
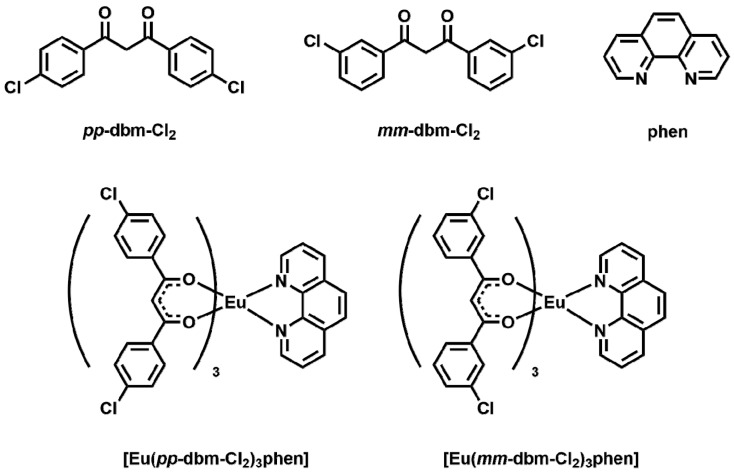
Compounds studied herein.

In this regard, in our study, we specifically incorporated halogens in our complex design to study the influence of halogens on both TL and AIE. Two chloride-containing ligands, *pp*-dbm-Cl_2_ and *mm*-dbm-Cl_2_ were employed for lanthanide(III) complexation, and to rule out the effect of ionic charge on TL, an auxiliary ligand 1,10-phenanthroline (phen) was used to complete the lanthanide coordination sphere, forming neutral Ln^III^ complexes (Figure 1). As Eu^III^ ions are known to be efficiently sensitized by β-diketonates, [Eu(*pp*-dbm-Cl_2_)phen] and [Eu(*mm*-dbm-Cl_2_)phen] are designed, synthesized, and their photo-physical and structural study are discussed herein.

## 2. Results and Discussion

### 2.1. Synthesis and Characterization

The ligands *pp*-dbm-Cl_2_ and *mm*-dbm-Cl_2_ were synthesized by a classical Claisen condensation reaction between the corresponding ketone and methyl ester using NaH as the base. Both compounds were purified by recrystallization using ethanol, with product yields of 47% and 27% for *pp*-dbm-Cl_2_ and *mm*-dbm-Cl_2_, respectively. These compounds were characterized by ^1^H-NMR, ^13^C-NMR, and CHN elemental analysis.

The corresponding complexes were synthesized by addition of europium(III) chloride to a mixture of auxiliary ligands and the deprotonated dbm-Cl_2_ by triethylamine at room temperature. Purification was performed by recrystallization with a solution mixture of THF and MeCN. Single crystals for X-ray diffraction experiments were obtained by slow evaporation of the above solvent mixture. These complexes were characterized by ^1^H-NMR, CHN elemental analysis and SCXRD.

**Figure 2:**
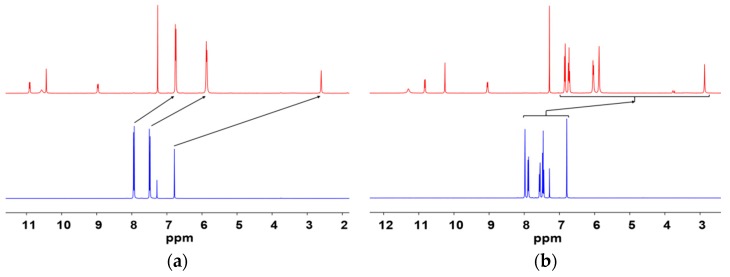
^1^H-NMR (400 Hz, CDCl_3_) spectra of (**a**) [Eu(*pp*-dbm-Cl_2_)_3_phen] (top) and *pp*-dbm-Cl_2_ (bottom) and (**b**) [Eu(*mm*-dbm-Cl_2_)_3_phen] (top) and *mm*-dbm-Cl_2_ (bottom).

The successful complexation can also be evidenced by ^1^H-NMR spectra in Figure 2. The spectra of the complexes show shifted dbm-Cl_2_ and phen peaks caused by the paramagnetic nature of Eu^III^ ions. Additionally, the 3:1 ratio of dbm-Cl_2_ and phen indicates the formation of [Eu(dbm-Cl_2_)_3_phen].

**Figure 3:**
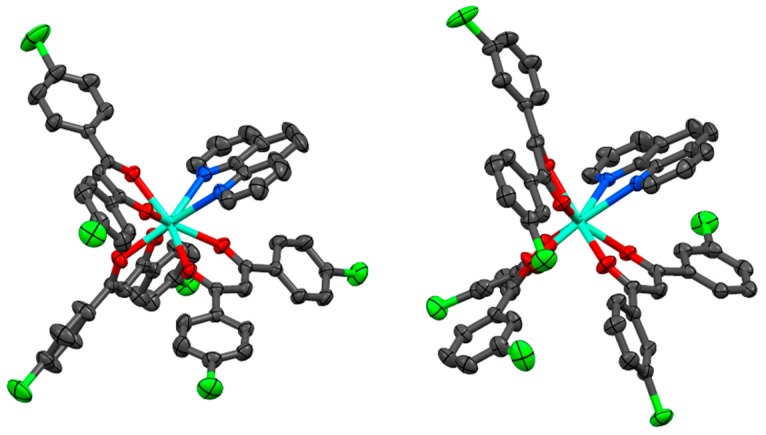
Crystal structures of [Eu(*pp*-dbm-Cl_2_)_3_phen] (left) and [Eu(*mm*-dbm-Cl_2_)_3_phen] (right) Hydrogen atoms are omitted for clarity. Thermal ellipsoids are drawn at 50% probability. Color code: cyan (Eu); red (O); blue (N); green (Cl); and black (C).

The molecular structures of the Eu^III^ complexes were revealed by X-ray diffraction measurement (Figure 3 and Table S1). For complex [Eu(*pp*-dbm-Cl_2_)_3_phen], the metal centres are surrounded by three *pp*-dbm-Cl_2_ and one phen with a coordination number of eight. The coordination geometry can be examined by the use of a Shape Analysis program developed by Raymond’s group [16,17]. The structures can be best described as having a bicapped trigonal prism (*C*_2*v*_) geometry. The bond lengths of Eu–O range from 2.326 to 2.356 Å, and those of Eu-N are 2.605 and 2.617 Å. The average O-Eu-O and N-Eu-N bond angles are 70.8 ± 0.1 and 62.89°, respectively.

For [Eu(*mm*-dbm-Cl_2_)_3_phen], the coordination is similar to [Eu(*pp*-dbm-Cl_2_)_3_phen] with three *mm*-dbm-Cl_2_ and one phen. Shape analysis indicates the coordination geometry fits the bicapped trigonal prism as well. The bond lengths of Eu-O range from 2.318 to 2.382 Å and those of Eu-N are 2.582 and 2.645 Å. The average O-Eu-O and N-Eu-N bond angles are 70.5 ± 0.6 and 62.68°.

Compared to the similar structure of [Eu(dbm)_3_phen] reported previously [4], these two complexes show normal bond lengths and angles, but the coordination geometry is, however, different. [Eu(dbm)_3_phen] was best fitted to a square antiprism (*D*_4*d*_) geometry. We hypothesize that this difference arises due to the presence of C-H···Cl-C and π···Cl interactions in both the [Eu(*pp*-dbm-Cl_2_)_3_phen] and [Eu(*mm*-dbm-Cl_2_)_3_phen] structures.

### 2.2. Luminescent Properties

**Figure 4:**
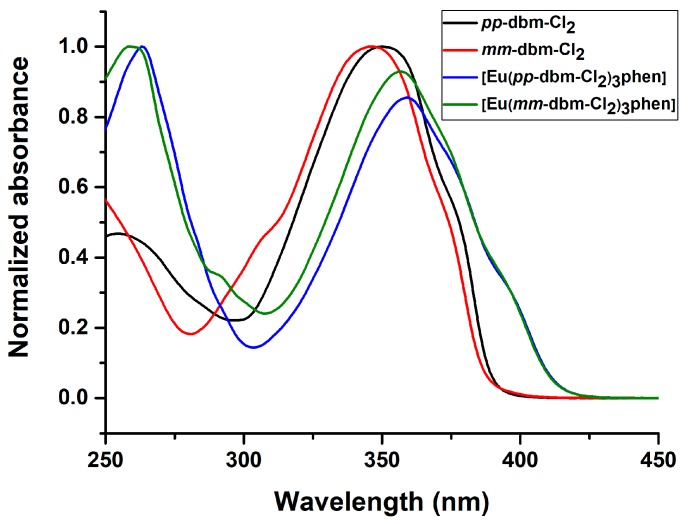
Normalized UV-vis absorption spectra of *pp*-dbm-Cl_2_, *mm*-dbm-Cl_2_, [Eu(*pp*-dbm-Cl_2_)_3_phen], and [Eu(*mm*-dbm-Cl_2_)_3_phen] in THF, 10 µM.

The UV-vis absorption spectra of the ligands, *pp*-dbm-Cl_2_ and *mm*-dbm-Cl_2_ and their corresponding Eu complexes are recorded in THF solution and shown in Figure 4. For *pp*-dbm-Cl_2_, the peak maximum is at 350 nm which is attributed to the π → π* transition. For *mm*-dbm-Cl_2_, this is at 347 nm, which is slightly more blue-shifted than that of *pp*-dbm-Cl_2_. This is attributed to the less symmetrical structure of *mm*-dbm-Cl_2_. Upon complexation, [Eu(*pp*-dbm-Cl_2_)_3_phen] shows absorption at 360 nm, which is red-shifted 10 nm compared to *pp*-dbm-Cl_2_. The complexation with Eu^III^ ion lowers the energy of the ligand. The peak at 270 nm is due to the π → π* transition of phen. [Eu(*mm*-dbm-Cl_2_)_3_phen] exhibits the same trend with an absorption peak maximum at 357 nm.

**Figure 5:**
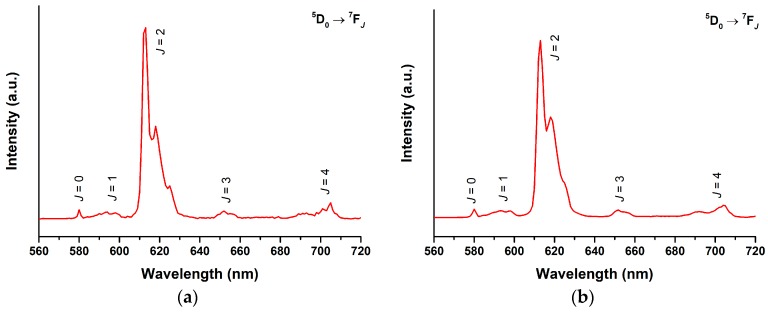
Photoluminescence spectra of (**a**) [Eu(*pp*-dbm-Cl_2_)_3_phen] and (**b**) [Eu(*mm*-dbm-Cl_2_)_3_phen] in THF, 10 µM. Measurement condition: λ_ex_ = 360 nm, longpass filter = 380 nm.

The photoluminescence properties of the Eu^III^ complexes in THF solution were studied (Figure 5). The observed emission spectra shows a typical fingerprint Eu^III^ emission profile with ^5^D_0_ → ^7^F*_J_* (*J* = 0–4) transitions upon excitation at 360 nm. Excitation spectra for the ^5^D_0_ → ^7^F_2_ peak (612 nm) shows a broad band with peak maximum at 360 nm, which is similar to their absorption spectrum, indicating that the luminescence is mainly originated from the absorption of *pp*-dbm-Cl_2_ or *mm*-dbm-Cl_2_ (Figures S7 and S8). The symmetry of the Eu^III^ centre can be evaluated by calculating the asymmetric ratio, which is the intensity ratio between the *J* = 2 and *J* = 1 transition. The values are 19.4 and 17.2, indicating a low symmetry in the Eu^III^ site, which is in agreement with the crystal structure. Furthermore, the luminescence decay of the ^5^D_0_ → ^7^F_2_ transition were best fitted to be biexponential (Figures S9 and S10), which indicates the presence of some coordinating solvent molecules. The resulting lifetimes are 25.1 (93%) and 115.8 (7%) µs for [Eu(*pp*-dbm-Cl_2_)_3_phen], and 11.7 (75%) and 65.4 (25%) µs for [Eu(*mm*-dbm-Cl_2_)_3_phen]. Their quantum yield is only 0.4% and 0.03% in THF solution at 10 µM for [Eu(*pp*-dbm-Cl_2_)_3_phen] and [Eu(*mm*-dbm-Cl_2_)_3_phen], respectively. Such low values are seemingly attributed to inefficient energy transfer from *pp*-dbm-Cl_2_ or *mm*-dbm-Cl_2_ to the Eu^III^ centre, but this is not supported by the triplet values obtained from the low temperature phosphorescence measurements of [Gd(*pp*-dbm-Cl_2_)_3_phen] and [Gd(*mm*-dbm-Cl_2_)_3_phen], which are both 20,202 cm^−1^ (Figures S11 and S12). These values are slightly higher than the lowest excited state of Eu^III^ ions ^5^D_0_ at 17,200 cm^−1^, which should promote the efficient energy transfer process [9]. The weak luminescence may be due to quenching by solvent molecules because at solid state, both Eu^III^ complexes are luminescent, visible by the naked eye. This is also suggested from the biexponential lifetimes obtained.

**Figure 6:**
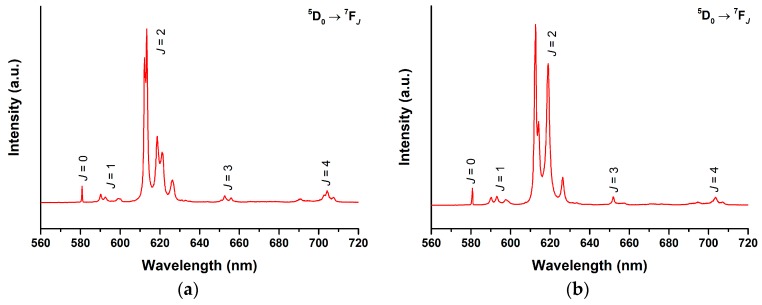
Solid-state photoluminescence spectra of (**a**) [Eu(*pp*-dbm-Cl_2_)_3_phen] and (**b**) [Eu(*mm*-dbm-Cl_2_)_3_phen]. Measurement condition: λ_ex_ = 360 nm, slit = 0.5–0.1, and longpass filter = 380 nm.

The solid-state emission spectra of [Eu(*pp*-dbm-Cl_2_)_3_phen] and [Eu(*mm*-dbm-Cl_2_)_3_phen] were recorded (Figure 6). Since the intensity at solid state is higher than that in the solution state, the emission profiles show finer splitting of the Eu^III^ profile. [Eu(*pp*-dbm-Cl_2_)_3_phen] contains five peaks at the ^5^D_0_ → ^7^F_2_ transition, whereas [Eu(*mm*-dbm-Cl_2_)_3_phen] has four peaks, revealing low symmetry of the Eu^III^ site (C_2v_ and C_s_, respectively). Unlike the solution state, the luminescence decay of the ^5^D_0_ → ^7^F_2_ transition at solid state is fitted to a monoexponential decay and the resulting lifetimes are much longer, 455 and 317 µs for [Eu(*pp*-dbm-Cl_2_)_3_phen] and [Eu(*mm*-dbm-Cl_2_)_3_phen], respectively (Figures S13 and S14). The quantum yields are measured to be 73% and 38% for [Eu(*pp*-dbm-Cl_2_)_3_phen] and [Eu(*mm*-dbm-Cl_2_)_3_phen]. Such an increase is probably due to the rigidity of the solid that minimizes molecular motion, as well as eliminating the factor of solvent quenching to the inner and outer coordination sphere.

**Figure 7:**
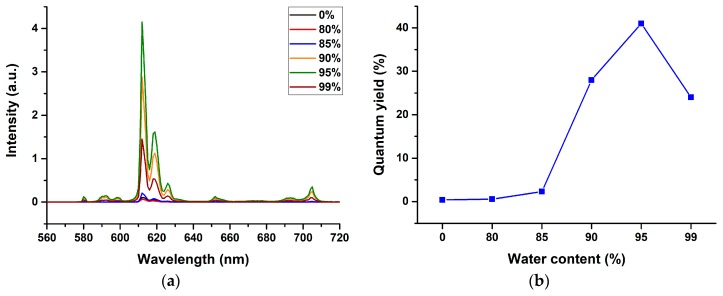
(**a**) Photoluminescence spectra of [Eu(*pp*-dbm-Cl_2_)_3_phen] in THF/water mixture at different water content (*f*_w_), Measurement condition: λ_ex_ = 360 nm, slit = 1.0–0.3, and longpass filter = 380 nm. (**b**) Trend of quantum yield of [Eu(*pp*-dbm-Cl_2_)_3_phen] in THF/water mixture at different water content.

Due to such dramatic differences in the luminescence properties, we postulated that these compounds might exhibit some aggregation-induced emission (AIE) properties. To illustrate this, we demonstrated the luminescence behavior of [Eu(*pp*-dbm-Cl_2_)_3_phen] in THF-water mixtures with various water fractions (*f*_w_) at 10 µM (Figure S15). By naked eye, the solution turns turbid at 80% water content, indicating the formation of aggregates. This can also be proved by the UV-vis absorption spectra because at 80% *f*_w_, a level-off tail appears, and the light transmission decreases due to Mie scattering, indicating aggregation. (Figure S16). The absorption band also becomes broader, resembling the absorption in the solid state (Figure S17), and the red-shifting indicates formation of J-aggregates. The luminescence of the aggregates are recorded, and their spectra are shown in Figure 7 and Figure S18 (with higher water content). From 0% to 70% *f*_w_, the emission intensity drops, as the aggregates have not yet been formed, and the quenching arises from the presence of the O-H oscillating phonons from the water, which quenches the Eu^III^ luminescence. When *f*_w_ increases from 80% to 95%, the luminescence intensity increases, and the quantum yield rises from 0.56% to 41%. This is due to the formation of a higher content of smaller hydrophobic aggregates that protect the Eu^III^ center from water. However, when the water content increases to 99%, the luminescence intensity drops (Ф = 24% at 99%) because the aggregate size becomes smaller (95% = 131 nm and 99% = 88 nm), lowering the number of [Eu(*pp*-dbm-Cl_2_)_3_phen] encapsulated in the aggregate. This phenomenon is similar to the study by Zhang et al. [18].

### 2.3. Tribolumimescence and Molecular Packing

**Figure 8:**
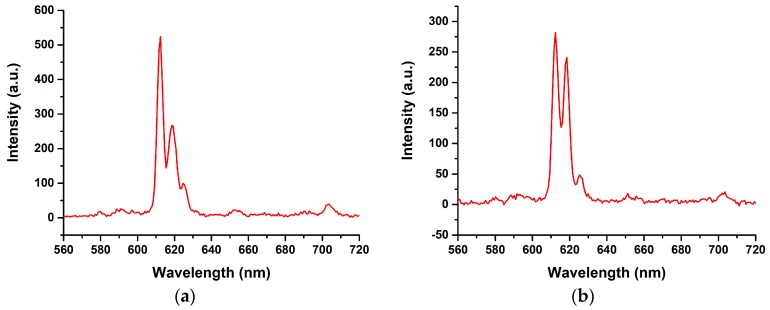
Triboluminescence spectra of (**a**) [Eu(*pp*-dbm-Cl_2_)_3_phen] and (**b**) [Eu(*mm*-dbm-Cl_2_)_3_phen].

From the findings of the AIE property, [Eu(*pp*-dbm-Cl_2_)_3_phen] is highly emissive with decent quantum yield in the solid state, and hence is ideal for triboluminescence study. Together with [Eu(*mm*-dbm-Cl_2_)_3_phen], their dried crystals were crashed by a glass rod and were both found to be TL-active, evident by the TL spectra shown in Figure 8. In fact, these TL lights are very difficult to be seen by naked eye because these TL are very weak compared to their PL; therefore, the TL spectra contains much noise. Nevertheless, the TL spectra are identical to their corresponding PL spectra, indicating that the symmetry of the complexes did not change upon fracture of the crystal structure. If so, the splitting of the hypertensive peaks would have been altered [19].

To correlate triboluminescence with its molecular structure, the crystal structures of [Eu(*pp*-dbm-Cl_2_)_3_phen] and [Eu(*mm*-dbm-Cl_2_)_3_phen] were studied. Both compounds share the same symmetry with a centrosymmetric space group *P*2_1_/*n*. According to Zink et al., non-centrosymmetric crystals are likely to exhibit TL because of the piezoelectric effect [20]. The weak TL of [Eu(*pp*-dbm-Cl_2_)_3_phen] and [Eu(*mm*-dbm-Cl_2_)_3_phen] may be due to the lack of piezoelectric charge generated due to their centrosymmetricity. This may explain why their TL is only detected when the intact crystal is fractured. When the crystal is crushed into much smaller grains, no TL signal can be detected. It is because, when fracture occurs in large crystals, the newly formed cracked surfaces have larger surface area in which more charge can be accumulated than for the smaller crystals. However, for the non-centrosymmetric structures such as [Eu(dbm)_4_TMP] reported previously [4], even when it is grinded to a crystalline powder, there is still detectable TL generated upon mechanical action.

**Figure 9:**
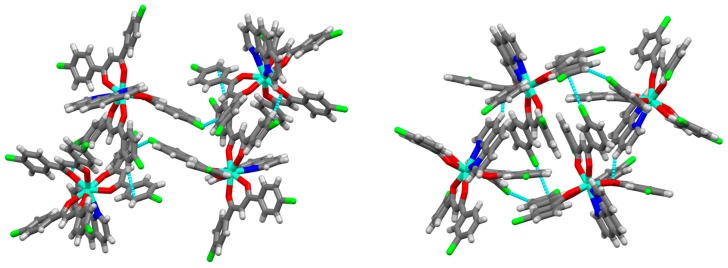
Unit cell of [Eu(*pp*-dbm-Cl_2_)_3_phen] (**left**) and [Eu(*mm*-dbm-Cl_2_)_3_phen] (**right**).

We also observed that the molecular packing within the crystal structure also has an influence on the TL. With a change in the Cl position, the packing mode was found to differ slightly for the two complexes (Figure 9). For the Cl at the para position, [Eu(*pp*-dbm-Cl_2_)_3_phen], one complex is connected to five others by different intermolecular interaction. There are π···π interactions (3.607, 3.660, 3.734 Å), C-H···π interactions (2.625 Å), C-H···O interactions (2.441 Å) and C-H···Cl-C (2.788 Å). For [Eu(*mm*-dbm-Cl_2_)_3_phen], each complex is surrounded by eight more complexes, connected through Van der Vaals forces. There are π···π interactions, C-H···O interactions (2.496 Å) and C-H···Cl-C (2.861, 2.883 Å) and C-Cl···π (3.448 Å). When Cl is at the meta position, all the C-H···π interactions are changed to a weaker C-Cl···π interactions (longer distance). This may decrease the rigidity of the crystal causing more slippage upon mechanical forces, hence accounting for the poorer luminescence quantum yield and much weaker TL observed for [Eu(*mm*-dbm-Cl_2_)_3_phen] than [Eu(*pp*-dbm-Cl_2_)_3_phen].

## 3. Experimental

### 3.1. Material Preparation

Unless otherwise stated, all reagents for synthesis were obtained commercially and used without further purification. Methyl 4-chlorobenzoate, 4′-chloroacetophenone, methyl 3-chlorobenzoate, and 3′-chloroacetophenone were purchased from Meryer (Shanghai, China). Sodium hydride, 1,10-phenanthroline, europium(III) chloride hexahydrate (99.99%), gadolinium(III) chloride hexahydrate (99.999%) were purchased from Sigma (St. Louis, MO, USA). Anhydrous tetrahydrofuran was purchased from Acros (Waltham, MA, USA). The ligand synthesis was performed using standard Schlenk line techniques. Glassware was dried at 130 °C prior use for water-free reaction.

### 3.2. Synthesis of Ligands and Complexes

*1,3-Bis(4-chlorophenyl)propane-1,3-dione (pp-dbm-Cl_2_).* To a 500 mL two-neck round bottom flask were added sodium hydride (60% in mineral oil, 5.0 g, 125 mmol) and then anhydrous THF (200 mL). This mixture was cooled to 0 °C, and then methyl 4-chlorobenzoate (55 mmol) and 4’-chloroacetophenone (50 mmol) were added. The suspension was heated to reflux under nitrogen for 48 h. After cooled down to room temperature, the mixture was filtered through Celite and washed once with ethanol. The filtrate was treated with a mixture of ether and dilute hydrochloric acid. The ether phase was separated and washed three times with brine and dried on magnesium sulfate. After removal of the ether, the residue was recrystallized by ethanol to give pure products as white or light-yellow solids. Yield 47%. ^1^H-NMR (400 MHz, CDCl_3_) δ 16.75 (s, 1H), 7.91 (d, *J* = 12 Hz, 4H), 7.46 (d, *J* = 8 Hz, 4H), 6.76 (s, 1H). ^13^C-NMR (100 MHz, CDCl_3_) δ 184.60, 138.92, 133.76, 129.04, 128.53, and 92.88. Anal. (%) Calcd for C_15_H_10_Cl_2_O_2_: C, 61.46; and H, 3.44. Found: C, 61.48 and H, 3.36.

*1,3-bis(3-chlorophenyl)propane-1,3-dione (mm-dbm-Cl_2_)*. The procedure is the same as *pp*-dbm-Cl_2_ by using reagents 3-chlorobenzoate and 3′-chloroacetophenone instead. Yield 27%. ^1^H-NMR (400 MHz, CDCl_3_) δ 16.61 (s, 1H), 7.95 (s, 2H), 7.86 (d, *J* = 7.8 Hz, 2H), 7.53 (d, *J* = 8.8 Hz, 2H), 7.44 (t, *J* = 7.9 Hz, 2H), 6.77 (s, 1H). ^13^C-NMR (100 MHz, CDCl_3_) δ 184.49, 137.04, 135.02, 132.54, 130.03, 127.33, 125.30, and 93.45. Anal. (%) Calcd for C_15_H_10_Cl_2_O_2_: C, 61.46; and H, 3.44. Found: C, 61.47 and H, 3.32.

*[Eu(pp-dbm-Cl_2_)_3_phen]*. To a 10 mL reaction tube with *pp-dbm-Cl_2_* (0.3 mmol) and 1,10-phenanthroline (0.1 mmol) was added 1 mL THF, triethylamine (0.3 mmol) and 1 mL of 0.1 M europium(III) chloride hexahydrate ethanolic solution. The mixture was stirred overnight. The solvent was evaporated, and the solid was washed with water and MeCN. Recrystallization of the solid with THF/MeCN mixture yielded a yellow crystalline solid. The same approach was applied for all the Ln^III^ complexes in this study. Yield: 75%. ^1^H-NMR (400 MHz, CDCl_3_) δ 10.91 (d, *J* = 7.7 Hz, 2H), 10.57 (s, 2H), 10.44 (s, 2H), 8.97 (d, *J* = 7.5 Hz, 2H), 6.75 (d, *J* = 7.5 Hz, 12H), 5.87 (d, *J* = 7.7 Hz, 12H), and 2.60 (s, 3H). Anal. (%) Calcd for C_57_H_35_Cl_6_N_2_O_6_Eu: C, 56.65; H, 2.92; and N, 2.32. Found: C, 56.67; H, 2.85; and N, 2.25.

*[Eu(mm-dbm-Cl_2_)_3_phen]*. Yield: 74%. ^1^H-NMR (400 MHz, CDCl_3_) δ 11.27 (s, 2H), 10.80 (d, *J* = 7.7 Hz, 2H), 10.23 (s, 2H), 9.02 (d, *J* = 7.7 Hz, 2H), 6.82 (d, *J* = 7.9 Hz, 6H), 6.70 (t, *J* = 7.7 Hz, 6H), 6.01 (d, *J* = 7.3 Hz, 6H), 5.85 (s, 6H), and 2.85 (s, 3H). Anal. (%) Calcd for C_57_H_35_Cl_6_N_2_O_6_Eu: C, 56.65; H, 2.92; and N, 2.32. Found: C, 56.69; H, 2.86; and N, 2.24.

### 3.3. Characterization Measurement

NMR spectra were recorded on a Bruker Ultrashield Advance Pro 400 spectormeter (Billerica, MA, USA), and the chemical shifts were referenced internally to tetramethylsilane or the corresponding solvents residue in parts per million (ppm). FT-IR spectra were recorded on a Nicolet iS 50 FT-IR spectrometer (Waltham, MA, USA) with a KBr pellet. Elemental analysis was performed on an Elementar Vario Micro Cube elemental analyzer (Langenselbold, Germany). Dynamic light scattering experiment to measure the size distributions of the aggregates were on the Zetasizer Nano ZS (Malvern, UK).

### 3.4. Single Crystal Structure Determination

Single crystals of the Eu^III^ complexes were grown by slow evaporation of THF/MeCN mixture. A suitable crystal was picked and mounted using the “oil-drop mounting” technique and its data were collected using either the Bruker Smart Apex II or Bruker D8-Venture single-crystal diffractometer (Madison, WI, USA). Multiscan absorption correction was then applied to the data using the Bruker SAINT (version 8.34A)/SADABS (ver. 2014/5) software (Billerica, MA, USA). The SHELX program suite (SHELX97 or SHELX 2014/7) was used to calculate the initial structural solution through either the direct or the Patterson method, and was then refined by full matrix least-squares on F2. CCDC 1890158, 1890142.

### 3.5. Photophysical Measurement

UV-vis absorption spectra were measured on an Agilent HP UV-8453 spectrophotometer (Santa Clara, CA, USA). Steady-state room temperature photoluminescence measurements were performed on an Edinburgh Instrument (Livingston, UK) FLSP920 spectrophotometer equipped with a Xe900 continuous xenon lamp, mF920 microsecond flashlamp and a single photon counting photomultiplier tube. Spectra were corrected with the bundled F900 software. Reflectance and absolute quantum yield were recorded by using a corrected integrating sphere provided by Edinburgh Instrument. Triboluminescence spectra were recorded on an Ocean Optics QEPro multichannel spectrometer (Largo, FL, USA) [4].

## 4. Conclusions

In summary, two novel Eu^III^ complexes were developed. The complexes are typical AIE material exhibiting strong luminescence in the solid state but being non-emissive in the solution state. Due to the centrosymmetric space group, these compounds are weakly TL-active, and hence the intensity of these compounds are not comparable with those of a non-centrosymmetric structure due to the lack of piezoelectric charge. With the introduction of a Cl group at the para position, [Eu(*pp*-dbm-Cl_2_)_3_phen] has higher quantum yield and displays a slightly more intense TL than [Eu(*mm*-dbm-Cl_2_)_3_phen] due to a better and more rigid packing that reduces non-radiative dissipation.

## Supplementary Materials

The following are available online at http://www.mdpi.com/1420-3049/24/4/662/s1, Figure S1–S17 and Table S1: Spectra of characterization and photophysical measurement, crystallographic details.
