# Supplementary material for: Triboluminescence of Centrosymmetric Lanthanide β-Diketonate Complexes with Aggregation-Induced Emission
Source: Molecules. 2019 Feb 13;24(4):662. doi: 10.3390/molecules24040662 (PMC6412301; doi:10.3390/molecules24040662)

checkCIF/PLATON report

Structure factors have been supplied for datablock(s) 1_a

THIS REPORT IS FOR GUIDANCE ONLY. IF USED AS PART OF A REVIEW PROCEDURE FOR PUBLICATION, IT SHOULD NOT REPLACE THE EXPERTISE OF AN EXPERIENCED CRYSTALLOGRAPHIC REFEREE.

No syntax errors found.   CIF dictionary   Interpreting this report

Datablock: 1_a

```
Bond precision:  C-C = 0.0042 A          Wavelength=0.71073

Cell:           a=13.8631(6)    b=16.6671(9)       c=22.7611(11)
                alpha=90        beta=105.529(2)    gamma=90
Temperature:    233 K

                Calculated                 Reported
Volume          5067.1(4)                  5067.1(4)
Space group     P 21/n                     P 21/n
Hall group      -P 2yn                     -P 2yn
Moiety formula  C57 H35 Cl6 Eu N2 O6       ?
Sum formula     C57 H35 Cl6 Eu N2 O6       C57 H35 Cl6 Eu N2 O6
Mr              1208.54                    1208.53
Dx,g cm-3       1.584                      1.584
Z               4                          4
Mu (mm-1)       1.609                      1.609
F000            2416.0                     2416.0
F000'           2419.72
h,k,lmax        17,20,28                   17,20,28
Nref            10387                      10366
Tmin,Tmax       0.555,0.824                0.656,0.745
Tmin'           0.415

Correction method= # Reported T Limits: Tmin=0.656 Tmax=0.745
AbsCorr = MULTI-SCAN

Data completeness= 0.998          Theta(max)= 26.390

R(reflections)= 0.0270( 8693)     wR2(reflections)= 0.0618( 10366)

S = 1.027                  Npar= 649
```

The following ALERTS were generated. Each ALERT has the format
test-name_ALERT_alert-type_alert-level.
Click on the hyperlinks for more details of the test.

Alert level C

```
PLAT241_ALERT_2_C High   'MainMol' Ueq as Compared to Neighbors of        O4 Check
PLAT241_ALERT_2_C High   'MainMol' Ueq as Compared to Neighbors of        O5 Check
PLAT910_ALERT_3_C Missing # of FCF Reflection(s) Below Theta(Min).         6 Note
PLAT911_ALERT_3_C Missing FCF Refl Between Thmin & STh/L=    0.600         6 Report
```

Alert level G

```
PLAT794_ALERT_5_G Tentative Bond Valency for Eu1       (III)     .      2.83 Info
PLAT912_ALERT_4_G Missing # of FCF Reflections Above STh/L=  0.600         9 Note
PLAT978_ALERT_2_G Number C-C Bonds with Positive Residual Density.         6 Info
```

0 **ALERT level A** = Most likely a serious problem - resolve or explain
0 **ALERT level B** = A potentially serious problem, consider carefully
4 **ALERT level C** = Check. Ensure it is not caused by an omission or oversight
3 **ALERT level G** = General information/check it is not something unexpected

0 ALERT type 1 CIF construction/syntax error, inconsistent or missing data
3 ALERT type 2 Indicator that the structure model may be wrong or deficient
2 ALERT type 3 Indicator that the structure quality may be low
1 ALERT type 4 Improvement, methodology, query or suggestion
1 ALERT type 5 Informative message, check

It is advisable to attempt to resolve as many as possible of the alerts in all categories. Often the minor alerts point to easily fixed oversights, errors and omissions in your CIF or refinement strategy, so attention to these fine details can be worthwhile. In order to resolve some of the more serious problems it may be necessary to carry out additional measurements or structure refinements. However, the purpose of your study may justify the reported deviations and the more serious of these should normally be commented upon in the discussion or experimental section of a paper or in the "special_details" fields of the CIF. checkCIF was carefully designed to identify outliers and unusual parameters, but every test has its limitations and alerts that are not important in a particular case may appear. Conversely, the absence of alerts does not guarantee there are no aspects of the results needing attention. It is up to the individual to critically assess their own results and, if necessary, seek expert advice.

Publication of your CIF in IUCr journals

A basic structural check has been run on your CIF. These basic checks will be run on all CIFs submitted for publication in IUCr journals (*Acta Crystallographica*, *Journal of Applied Crystallography*, *Journal of Synchrotron Radiation*); however, if you intend to submit to *Acta Crystallographica Section C* or *E* or *IUCrData*, you should make sure that full publication checks are run on the final version of your CIF prior to submission.

Publication of your CIF in other journals

Please refer to the *Notes for Authors* of the relevant journal for any special instructions relating to CIF submission.

PLATON version of 19/10/2018; check.def file version of 15/10/2018

Datablock 1_a - ellipsoid plot

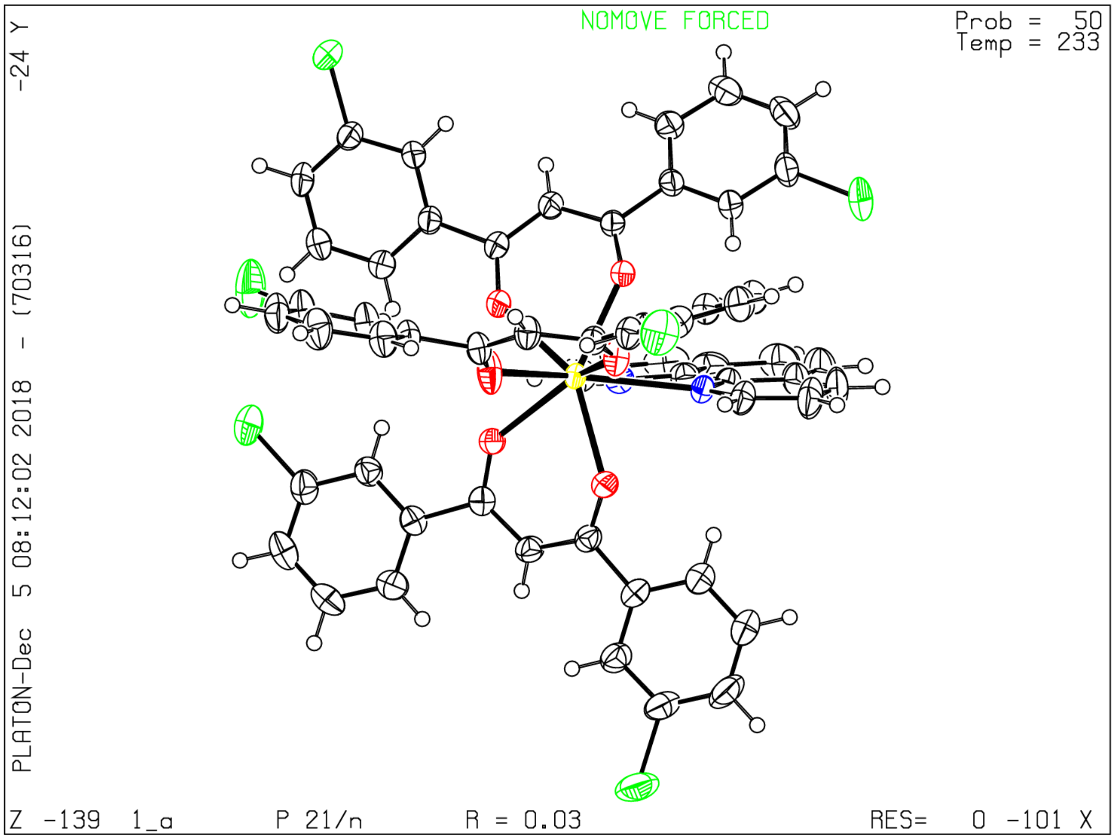

Supplement: Supplementary file 1 [file molecules-24-00662-s001.zip › SI-Molecules/[Eu(mm-dbm-Cl2)3phen] checkcif.pdf]
