# Supplementary material for: Triboluminescence of Centrosymmetric Lanthanide β-Diketonate Complexes with Aggregation-Induced Emission
Source: Molecules. 2019 Feb 13;24(4):662. doi: 10.3390/molecules24040662 (PMC6412301; doi:10.3390/molecules24040662)

# checkCIF/PLATON report

Structure factors have been supplied for datablock(s) WHY1809

THIS REPORT IS FOR GUIDANCE ONLY. IF USED AS PART OF A REVIEW PROCEDURE FOR PUBLICATION, IT SHOULD NOT REPLACE THE EXPERTISE OF AN EXPERIENCED CRYSTALLOGRAPHIC REFEREE.

No syntax errors found. CIF dictionary Interpreting this report

## Datablock: WHY1809

---

```
Bond precision:  C-C = 0.0038 A         Wavelength=0.71073

Cell:            a=14.203(4)     b=18.353(5)       c=20.347(6)
                 alpha=90        beta=93.656(8)    gamma=90
Temperature:     296 K

                Calculated                Reported
Volume          5293(3)                   5293(3)
Space group     P 21/n                    P 21/n
Hall group      -P 2yn                    -P 2yn
Moiety formula  C57 H35 Cl6 Eu N2 O6      C57 H35 Cl6 Eu N2 O6
Sum formula     C57 H35 Cl6 Eu N2 O6      C57 H35 Cl6 Eu N2 O6
Mr              1208.54                   1208.53
Dx,g cm-3       1.517                     1.517
Z               4                         4
Mu (mm-1)       1.540                     1.540
F000            2416.0                    2416.0
F000'           2419.72
h,k,lmax        18,24,26                  18,23,26
Nref            12634                     12234
Tmin,Tmax       0.656,0.735               0.672,0.748
Tmin'           0.643

Correction method= # Reported T Limits: Tmin=0.672 Tmax=0.748
AbsCorr = MULTI-SCAN

Data completeness= 0.968          Theta(max)= 27.870

R(reflections)= 0.0258( 10214)    wR2(reflections)= 0.0628( 12234)

S = 1.029                  Npar= 650
```

---

The following ALERTS were generated. Each ALERT has the format
**test-name_ALERT_alert-type_alert-level**.
Click on the hyperlinks for more details of the test.

## Alert level C

```
PLAT029_ALERT_3_C _diffrn_measured_fraction_theta_full value Low .      0.969 Why?
PLAT241_ALERT_2_C High   'MainMol' Ueq as Compared to Neighbors of         C6 Check
PLAT241_ALERT_2_C High   'MainMol' Ueq as Compared to Neighbors of        C12 Check
PLAT241_ALERT_2_C High   'MainMol' Ueq as Compared to Neighbors of        C14 Check
PLAT242_ALERT_2_C Low    'MainMol' Ueq as Compared to Neighbors of         C4 Check
PLAT242_ALERT_2_C Low    'MainMol' Ueq as Compared to Neighbors of         C7 Check
PLAT242_ALERT_2_C Low    'MainMol' Ueq as Compared to Neighbors of        C10 Check
PLAT242_ALERT_2_C Low    'MainMol' Ueq as Compared to Neighbors of        C13 Check
PLAT242_ALERT_2_C Low    'MainMol' Ueq as Compared to Neighbors of        C22 Check
PLAT242_ALERT_2_C Low    'MainMol' Ueq as Compared to Neighbors of        C37 Check
PLAT242_ALERT_2_C Low    'MainMol' Ueq as Compared to Neighbors of        C43 Check
PLAT334_ALERT_2_C Small Aver. Benzene C-C Dist C4       -C9              1.37 Ang.
PLAT334_ALERT_2_C Small Aver. Benzene C-C Dist C10      -C15             1.37 Ang.
PLAT910_ALERT_3_C Missing # of FCF Reflection(s) Below Theta(Min).          9 Note
PLAT911_ALERT_3_C Missing FCF Refl Between Thmin & STh/L=    0.600         30 Report
PLAT913_ALERT_3_C Missing # of Very Strong Reflections in FCF ....         16 Note
```

## Alert level G

```
PLAT005_ALERT_5_G No Embedded Refinement Details Found  in the CIF     Please Do !
PLAT794_ALERT_5_G Tentative Bond Valency for Eu1       (III)     .       2.92 Info
PLAT899_ALERT_4_G SHELXL97   is Deprecated and Succeeded by SHELXL       2018 Note
PLAT912_ALERT_4_G Missing # of FCF Reflections Above STh/L=  0.600        340 Note
PLAT978_ALERT_2_G Number C-C Bonds with Positive Residual Density.          4 Info
```

```
   0 ALERT level A = Most likely a serious problem - resolve or explain
   0 ALERT level B = A potentially serious problem, consider carefully
  16 ALERT level C = Check. Ensure it is not caused by an omission or oversight
   5 ALERT level G = General information/check it is not something unexpected

   0 ALERT type 1 CIF construction/syntax error, inconsistent or missing data
  13 ALERT type 2 Indicator that the structure model may be wrong or deficient
   4 ALERT type 3 Indicator that the structure quality may be low
   2 ALERT type 4 Improvement, methodology, query or suggestion
   2 ALERT type 5 Informative message, check
```

It is advisable to attempt to resolve as many as possible of the alerts in all categories. Often the minor alerts point to easily fixed oversights, errors and omissions in your CIF or refinement strategy, so attention to these fine details can be worthwhile. In order to resolve some of the more serious problems it may be necessary to carry out additional measurements or structure refinements. However, the purpose of your study may justify the reported deviations and the more serious of these should normally be commented upon in the discussion or experimental section of a paper or in the "special_details" fields of the CIF. checkCIF was carefully designed to identify outliers and unusual parameters, but every test has its limitations and alerts that are not important in a particular case may appear. Conversely, the absence of alerts does not guarantee there are no aspects of the results needing attention. It is up to the individual to critically assess their own results and, if necessary, seek expert advice.

**Publication of your CIF in IUCr journals**

A basic structural check has been run on your CIF. These basic checks will be run on all CIFs submitted for publication in IUCr journals (*Acta Crystallographica*, *Journal of Applied Crystallography*, *Journal of Synchrotron Radiation*); however, if you intend to submit to *Acta Crystallographica Section C* or *E* or *IUCrData*, you should make sure that full publication checks are run on the final version of your CIF prior to submission.

**Publication of your CIF in other journals**

Please refer to the *Notes for Authors* of the relevant journal for any special instructions relating to CIF submission.

---

**PLATON version of 23/04/2018; check.def file version of 23/04/2018**

**Datablock WHY1809** - ellipsoid plot

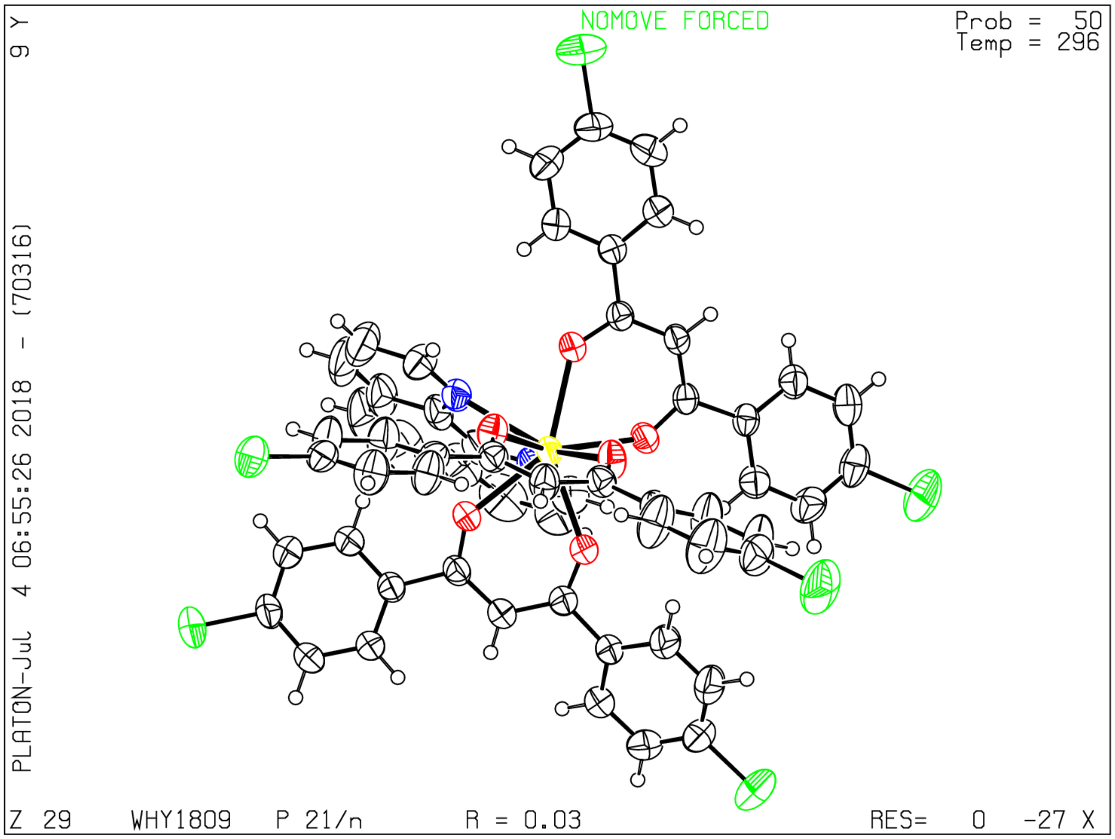

Supplement: Supplementary file 1 [file molecules-24-00662-s001.zip › SI-Molecules/[Eu(pp-dbm-Cl2)3phen] checkcif.pdf]
