# Supplementary material for: Triboluminescence of Centrosymmetric Lanthanide β-Diketonate Complexes with Aggregation-Induced Emission
Source: Molecules. 2019 Feb 13;24(4):662. doi: 10.3390/molecules24040662 (PMC6412301; doi:10.3390/molecules24040662)
Supplement: Supplementary file 1 [file molecules-24-00662-s001.zip › SI-Molecules/Supporting-GL-Molecules-Feb 2019-revised.docx]

**Supporting Information**

**Triboluminescence of centrosymmetric lanthanide β-diketonate complexes with aggregation-induced emission**

Ho-Yin Wong ^1^, Wesley Ting Kwok Chan ^1^ and Ga-Lai Law ^1,*^

Department of Applied Biology and Chemical Technology, Hong Kong Polytechnic University, Hung Hom, Hong Kong

*Correspondence: ga-lai.law@polyu.edu.hk; Tel.: +852-3400-8680

**Characterization and crystallography**

Figure S1. ^1^H NMR (400 Hz, CDCl_3_) spectra of *pp*-dbm-Cl_2_.

Figure S2. ^13^C NMR (100 Hz, CDCl_3_) spectra of *pp*-dbm-Cl_2_.

Figure S3. ^1^H NMR (400 Hz, CDCl_3_) spectra of *mm*-dbm-Cl_2_.

Figure S4. ^13^C NMR (100 Hz, CDCl_3_) spectra of *mm*-dbm-Cl_2_.

Figure S5. ^1^H NMR (400 Hz, CDCl_3_) spectra of [Eu(*pp*-dbm-Cl_2_)_3_phen].

Figure S6. ^1^H NMR (400 Hz, CDCl_3_) spectra of [Eu(*mm*-dbm-Cl_2_)_3_phen].

Table S1. Summary of crystal data and structure refinement.

| Compound | [Eu(*pp*-dbm-Cl_2_)_3_phen] | [Eu(*mm*-dbm-Cl_2_)_3_phen] |
| --- | --- | --- |
| Empirical formula | C_57_H_35_Cl_6_EuN_2_O_6_ | C_57_H_35_Cl_6_EuN_2_O_6_ |
| Formula weight | 1208.53 | 1208.53 |
| Temperature/K | 296(2) | 233(2) |
| Crystal system | monoclinic | monoclinic |
| Space group | *P*2_1_/*n* | *P*2_1_/*n* |
| a/Å | 14.2025(42) | 13.8631(6) |
| b/Å | 18.3534(52) | 16.6671(9) |
| c/Å | 20.3471(58) | 22.7611(11) |
| α/° | 90.00 | 90 |
| β/° | 93.6564(82) | 105.529(2) |
| γ/° | 90.00 | 90 |
| Volume/Å^3^ | 5293(3) | 5067.1(4) |
| Z | 4 | 4 |
| ρ_calc_g/cm^3^ | 1.517 | 1.584 |
| μ/mm^‑1^ | 1.540 | 1.609 |
| F(000) | 2416.0 | 2416.0 |
| Crystal size/mm^3^ | 0.28 × 0.28 × 0.20 | 0.54 × 0.31 × 0.12 |
| Radiation | MoKα (λ = 0.71073) | MoKα (λ = 0.71073) |
| 2Θ range for data collection/° | 4.88 to 55.74 | 4.446 to 52.78 |
| Index ranges | -18 ≤ h ≤ 18, -23 ≤ k ≤ 23, -25 ≤ l ≤ 26 | -16 ≤ h ≤ 17, -20 ≤ k ≤ 18, -28 ≤ l ≤ 25 |
| Reflections collected | 117171 | 42523 |
| Independent reflections | 12234 | 10366 |
| Data/restraints/parameters | 12234/0/650 | 10366/0/649 |
| Goodness-of-fit on F^2^ | 1.029 | 1.027 |
| Final R indexes [I>=2σ (I)] | R_1_ = 0.0258, wR_2_ = 0.0568 | R_1_ = 0.0270, wR_2_ = 0.0570 |
| Final R indexes [all data] | R_1_ = 0.0369, wR_2_ = 0.0628 | R_1_ = 0.0377, wR_2_ = 0.0618 |
| Largest diff. peak/hole / e Å^-3^ | 0.59/-0.45 | 0.42/-0.56 |

**Photophysical measurement**

Figure S7. Excitation spectrum of [Eu(*pp*-dbm-Cl_2_)_3_phen] in THF

Figure S8. Excitation spectrum of [Eu(*mm*-dbm-Cl_2_)_3_phen] in THF

Figure S9. Luminescence decay curve at ^5^D_0_ → ^7^F_2_ transition of [Eu(*pp*-dbm-Cl_2_)_3_phen] in THF, 10 μM. Red line: biexponential fit curve.

Figure S10. Luminescence decay curve at ^5^D_0_ → ^7^F_2_ transition of [Eu(*mm*-dbm-Cl_2_)_3_phen] in THF, 10 μM. Red line: biexponential fit curve.

Figure S11. Emission spectrum of [Gd(*pp*-dbm-Cl2)_3_phen] in 2-methyltetrahydrofuran at 77 K.

Figure S12 Emission spectrum of [Gd(*mm*-dbm-Cl_2_)_3_phen] in 2-methyltetrahydrofuran at 77 K.

Figure S13. Luminescence decay curve at ^5^D_0_ → ^7^F_2_ transition of [Eu(*pp*-dbm-Cl_2_)_3_phen] solid. Red line: monoexponential fit curve.

Figure S14. Luminescence decay curve at ^5^D_0_ → ^7^F_2_ transition of [Eu(*mm*-dbm-Cl_2_)_3_phen] solid. Red line: monoexponential fit curve.


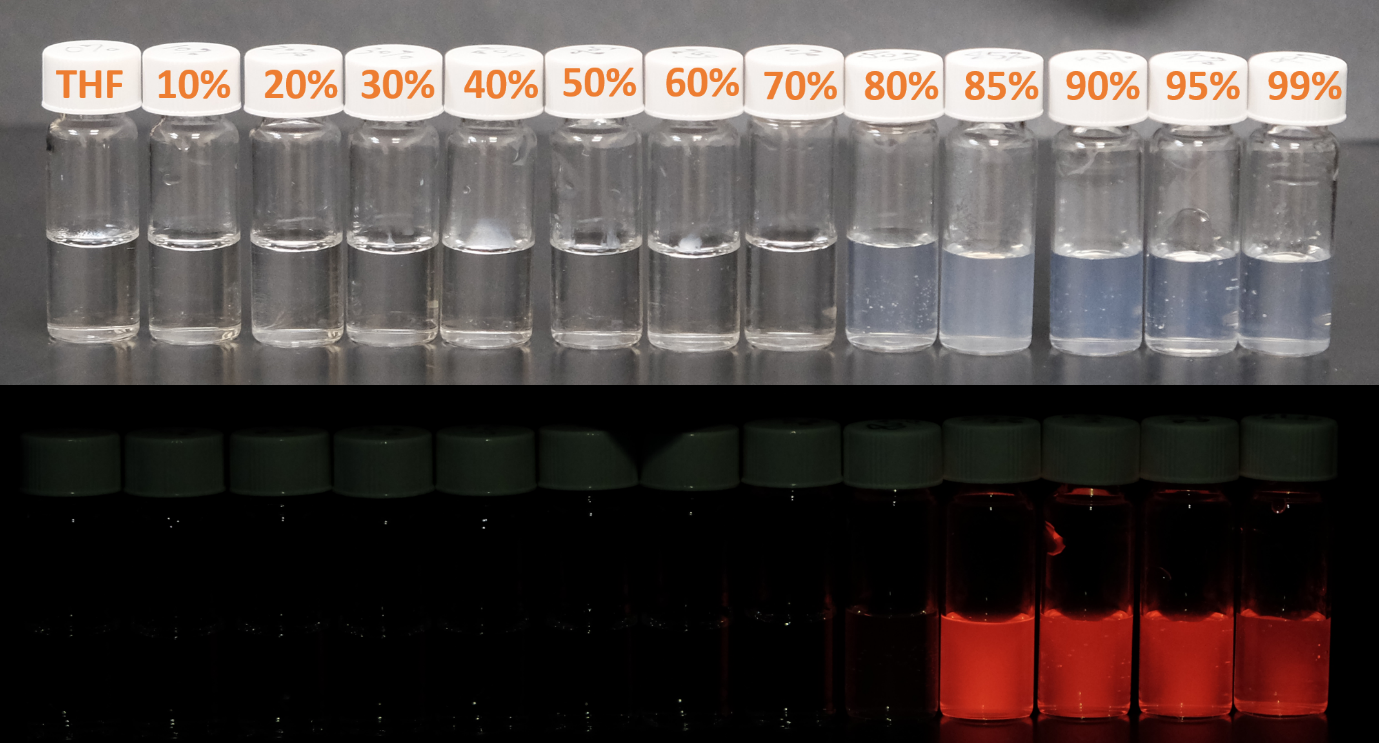


Figure S15. Image of [Eu(pp-dbm-Cl_2_)_3_phen] in THF/water with different water content.

Figure S16. UV-vis absorption spectra of [Eu(*pp*-dbm-Cl_2_)_3_phen] in THF/water mixture with different water content, 10 μM.

Figure S17. UV-vis absorption spectrum of [Eu(*pp*-dbm-Cl_2_)_3_phen] solid.

Figure S18. Photoluminescence spectra of [Eu(*pp*-dbm-Cl_2_)_3_phen] in THF/water mixture at different water content (*f*_w_), Measurement condition: *λ*_ex_ = 360 nm, slit = 1.0–0.3, longpass filter = 380 nm.

Figure S19. Excitation spectrum of [Eu(*pp*-dbm-Cl_2_)_3_phen] solid. Measurement condition: *λ*_em_ = 613 nm, slit = 0.5–0.1, longpass filter = 380 nm.

Figure S20. Excitation spectrum of [Eu(*mm*-dbm-Cl_2_)_3_phen] solid. Measurement condition: *λ*_em_ = 613 nm, slit = 0.5–0.1, longpass filter = 380 nm.
